# Symptoms of Poisoning with Whiskey

**Published:** 1841-09

**Authors:** 


### SYMPTOMS OF POISONING WITH WHISKEY.

Dr. J. Sweeny of Dripping Spring, Kentucky, was called to a boy 10 years old, who, several hours before, had drunk, at least a pint of whiskey. His prominent symptoms were, deep coma, with the eyelids partly open; the conjunctiva engorged, the pupils dilated and fixed, even in a strong light; inability to swallow; flushed cheeks; a discharge of frothy mucus from the mouth, tinged with blood; and violent convulsions. Death occurred without a subsequent examination of the body being made. D.

